# The preparation and relative bioavailability of an artemisin in self-emulsifying drug delivery system

**DOI:** 10.1080/10717544.2023.2168794

**Published:** 2023-01-28

**Authors:** Sijia Gao, Jingcai Chen, Wanqian Peng, Yang Yang, Yong Yang, Lei Hua, Yanlei Guo, Yunhong Wang, Xiaomei Zhang

**Affiliations:** a Chongqing Academy of Chinese Materia Medica, Chongqing, China; b School of Pharmacy, Chengdu University of TCM, Chegndu, China; c School of Pharmacy, Jiangxi University of Chinese Medicine, Jiangxi, China

**Keywords:** Artemisinin, self-emulsifying drug delivery systems (SEDDS), ternary phase diagram, central composite design-response surface methodology, bioavailability

## Abstract

The aim of this study is to demonstrate a method for improving the solubility and relative bioavailability of artemisinin using a self-emulsifying drug delivery system (SEDDS). The self-emulsifying drug load, solubility, and emulsifying time were used as the evaluation indices, based on a solubility test and a ternary phase diagram. Optimal Mixture Design in Design-Expert software was used to optimize the prescription of the artemisinin SEDDS. By determining the water distribution coefficient *in vitro,* combined with the drug concentration–time curve *in vivo*, a comparison was made of the relative oral bioavailability of the artemisinin SEDDS and the crude drug. The optimal prescription ratio of oleic acid polyethylene glycol glyceride, polyoxyethylene hydrogenated castor oil, and diethylene glycol monoethyl ether in the artemisinin SEDDS was 0.5:0.2:0.3 (wt/wt/wt), with a drug loading capacity of 41.556 mg/g, a solubility of 1.997 mg/mL, and a self-emulsification time of 214 s. The optimal prescription was transparent, slightly yellow, and oil-like. The average loading capacity of artemisinin was 41.912 mg/g, the emulsification time was 231 s, the average particle size was 128.0 nm, the average Zeta potential was –4.29 mV, and the solubility of artemisinin SEDDS in water was 1.997 mg mL^–1^. It is 33.85 times of the solubility of artemisinin in water, which achieves the purpose of increasing the solubility of artemisinin. The comparison of the oil/water distribution coefficient of the artemisinin SEDDS with that of the crude drug *in vitro* showed that SEDDS could improve the permeability of artemisinin and promote the absorption *in vivo*, and the relative bioavailability of the SEDDS agent was at least 1.47 times higher than that of the crude drug. The artemisinin SEDDS could significantly improve the solubility and relative bioavailability of artemisinin.

## 1. Introduction

A self-emulsifying drug delivery system (SEDDS) contains an isotropic mixture of oil phases, surfactants, co-surfactants, and drugs (Gurram et al., 2015). When exposed to water-soluble media, oil-in-water emulsion droplets can be rapidly formed by mild agitation or digestive movement in the gastrointestinal tract (Gursoy & Benita, 2004). SEDDS can be divided into self-micro-emulsifying drug delivery systems and self-nanoemulsifying drug delivery systems, according to the size of the self-emulsified droplets (Kohli et al., 2010). The numerous advantages of SEDDSs, including their physical stability, simple manufacturing process, and oral application via soft or hard gelatin capsules explain why they have been researched intensively within the last few decades (Gupta et al., 2013; Yeom et al., 2017; Komesli et al., 2019; Fidan et al., 2022). A renowned alternative approach for the delivery of drugs with low water solubility is using lipid formulations, particularly SEDDS, that deal with low aqueous solubility and poor oral bioavailability (Almeida & Tippavajhala, 2019).

Artemisinin is a compound with antimalarial activity isolated from *Artemisia annua*, a traditional Chinese medicine. It has the unique structure of a sesquiterpene lactone, which contains a peroxide bridge and has shown good therapeutic effects for all types of malaria, especially drug-resistant malaria. Previous research has shown that artemisinin causes cognitive impairment in diabetic mice (Qiu et al., 2022), kidney disease (Su-zhi et al., 2022), and atherosclerosis (Hu, 2022).

Artemisinin is a colorless needle-like crystal with the molecular formula of C_15_H_22_O_5_ and a molecular weight of 282.34 g/mol. It is soluble in ethyl acetate, chloroform, acetone, benzene, glacial acetic acid, diethyl ether, petroleum ether, ethanol, and methanol. Artemisinin is a sesquiterpene lactone with a peroxy bond and a delta-lactone ring with a 1,2,4-trioxane structural unit that includes peroxides. It has very low water solubility. Therefore, it is almost insoluble in water and has a melting point of 156–157 °C (Martino et al., 2019). The solubility in water was tested as 0.059 mg/mL by us. Artemisinin is a highly effective drug, especially for long-term malaria control, but its development has been limited due to its poor water solubility (Zhang et al., 2015). The present study describes a method to improve its solubility and relative bioavailability by means of a SEDDS.

## 2. Instruments and drug tests

### 2.1. Instruments

The following instruments were used: a high-performance liquid chromatography detector (Waters 2695-2998), an online degasser and quatpump (Waters Co.), BS-224S electronic scales (Sartorius, Germany), an Anke TGL-16 C high-speed centrifuge (Shanghai Anting Scientific Instrument Factory), an SK-1 quick mixer (Jintan Instrument Factory), an HZ-881S desktop water bath thermostatic oscillator (Jiangsu Taicang Experimental Equipment Factory); a DF-101S heating magnetic stirring apparatus (Eppendorf), and an RC-806 dissolution tester (Tianjin Tianda Tianfa Technology Co. Ltd.).

### 2.2. Drug test instruments and reagents

The following were obtained for the drug tests: artemisinin reference substance (National Institute for Food and Drug Control, China, batch number 100202), artemisinin bulk pharmaceutical chemicals (Chongqing Wulingshan KPC Pharmaceuticals Inc., batch number C00120170701, content 99.5%), oleic acid polyethylene glycol glyceride (Oleoyl Macrogolglycerides, batch number M01GS147525, Yuanye Bio-Technology Co. Ltd.), polyoxyethylene hydrogenated castor oil (Cremophor RH40, batch number Y23M10S83793, Shanghai Yuanye Bio-Technology Co. Ltd.), and Transcutol P (TP, batch number 177546, Tianrun Pharmaceutical Co.). The methanol was chromatographically pure, and the rest were analytically pure.

## 3. Methods and results

### 3.1. The determination of the artemisinin content

#### 3.1.1. The chromatographic conditions

High-performance liquid chromatography was used for the determination (Wu et al., 2021). The conditions were as follows: chromatographic column Target C18(2) (250 × 4.6 mm, 5 μm); moving phase acetonitrile–phosphate buffer solution (1.36 g of monopotassium phosphate was dissolved in 900 mL of water, the pH value was adjusted to 3.0 by the addition of phosphoric acid, and water was added to yield a final volume of 1000 mL) (44:56); detection wavelength 216 nm; flow velocity 1 mL/min; column temperature 35 °C; and sample injection content 10 μL.

#### 3.1.2. Preparation of the reference solution

An appropriate amount of artemisinin reference control was weighed out and dissolved in acetonitrile, and the solution was then shaken thoroughly.

#### 3.1.3. Preparation of the test solution

Artemisinin SEDDS was weighed out to 0.09 ± 0.01 g and placed in a 5-mL measuring bottle. To determine the drug load by external standard method, artemisinin in SEDDS needs to be dissolved. SEDDS are sticky, so it is difficult to operate if the sample size is too low, while the sample size is too large to prevent errors caused by incomplete dissolution. Compared with the loading of artemisinin on different SEDDS, the content determination results were more accurate when the sample size was more consistent. Therefore, artemisinin SEDDS were measured to 0.09 g. The preparation was then dissolved in acetonitrile and diluted to scale. The solution was then shaken for further detection.

#### 3.1.4. Preparation of the blank solution

Blank SEDDS without artemisinin was weighed out to 0.09 ± 0.01g and put into a 5-mL measuring bottle. The preparation was dissolved in acetonitrile, diluted to scale, and shaken vigorously.

#### 3.1.5. The specificity inspection

**Figure 1.**
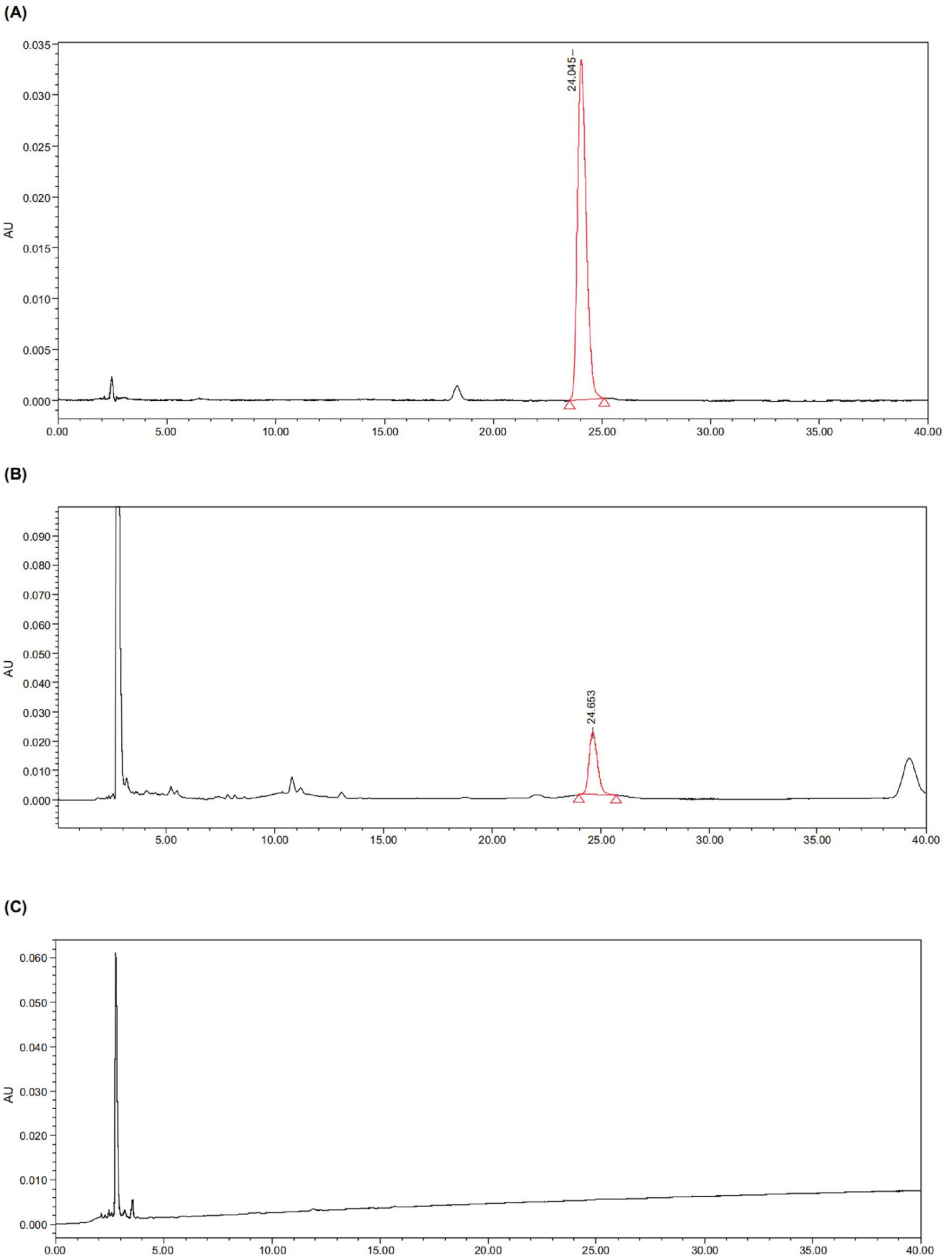
HPLC chromatograms of the blank solution (A), artemisinin reference solution (B), and test solution (C). A was the chromatogram of the artemisinin reference substance; the peak time of artemisinin was about 29 min. C was the blank solution with no chromatographic peak at 29 min. B had a corresponding chromatographic peak at about 29 min. This indicates that the matrix did not interfere with the detection of artemisinin.

Extracts of the blank solution, artemisinin control solution, and test solution were taken, and a determination of the artemisinin content was made in accordance with the above chromatographic conditions (***Figure 1***). As shown in Figure 1, the ingredients did not interfere with the detection of the artemisinin.

#### 3.1.6. The investigation of linear correlation

The artemisinin control solution was used to prepare a series of control solutions for the peak area measurement and recording. Linear regression was conducted with the mass concentration of the reference substance (μg/mL) as the x-coordinate (*x*) and the peak area as the y-coordinate (*y*) and was forced to go through the far point. The standard curve equation was *y* = 317.42*x*, *r* = 0.99999. The results showed that the concentration of artemisinin had a good linear correlation with the peak area within the range of 150.76–3015.2 μg/mL.

#### 3.1.7. Other methodology tests

A precision, stability, repeatability, and sample recovery test of the reference solution and test solution were conducted within 24 h. All the parameters met the requirements, indicating that the method was accurate. Both the reference solution and the test solution were stable for 24 h.

### 3.2. Design and optimization of artemisinin SEDDS prescription and validation of the optimal prescription

#### 3.2.1. Screening of the blank self-emulsification prescription

##### 3.2.1.1. The determination of the solubility of artemisinin in various solutions

Approximately 2 mL of different oil phases, surfactants, and co-surfactants were put into graduated tubes with stoppers. Extra amounts of crude artemisinin were added, and the tubes were vortexed. The tubes were then shaken in a constant temperature oscillation box at 37 °C for 24 h and centrifuged at 10,000 rpm for 10 min. The test solution was prepared as aforementioned, and the solubility of artemisinin in the various excipients was measured (***Table 1***).

**Table 1.** Solubility of artemisinin in different oil phases, emulsifiers, and co-emulsifiers (*n* = 3).

Oil phases	Solubility (mg g^–1^)	Surfactant	Solubility (mg g^–1^)	Co-surfactant	Solubility (mg g^–1^)
Medium chain triglyceride	13.279	Tween-20	3.059	glycerin	15.466
Glyceryl Monooleate	2.152	Tween-40	5.584	Polyethylene glycol	12.493
Soybean oil	7.551	Tween-60	2.731	Diethylene glycol monoethyl ether	30.529
Corn oil	3.057	Tween-80	2.303	Diethylene glycol monoethyl ether	55.70899
Olive oil	3.522	Span-80	1.535	1,2-propylene glycol	4.254
Castor oil	1.823	Isopropyl myristate	11.74	Isopropanol	27.726
Ethyl oleate	16.106	Triethanolamine	4.235		
Oleic acid polyethylene glycol glyceride	28.4126	Span-85	2.956		
Oleic acid	7.388	Polyethylene glycol monooleate	3.911		
		Polyoxyethylene castor oil	7.933		
		isopropyl palmitate	5.973		
		Oleoyl Macrogolglycerides	14.99272		
		Polyoxyethylene-7- stearate	2.349176		
		Polyoxyethylene hydrogenated castor oil	9.3628		
		Caprylic acid capric acid polyethylene glycol glyceride	309.3397		

##### 3.2.1.2. The drawing of a ternary phase diagram

**Figure 2.**
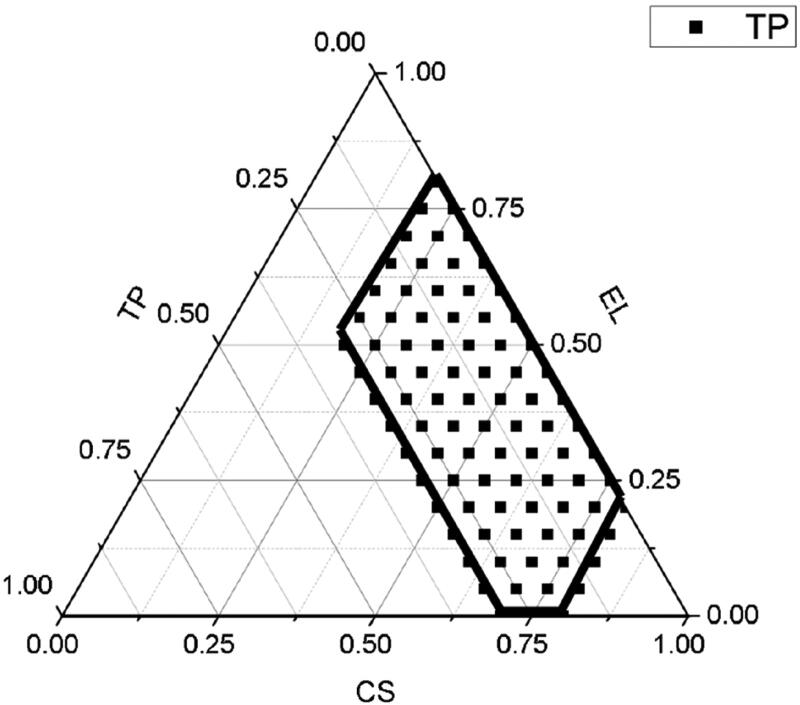
Ternary phase diagram of oil phase, surfactant, and cosurfactant in accordance with different sample proportions. The area that could form milk is circled as the forming area.

In the present study, the screening range of each phase in the SEDDS was limited as follows: 20%–80% for the oil phase, 20%–80% for the surfactant, and 0%–30% for the co-surfactants. Based on the above composition ranges, different proportions of the oil phase, surfactants, and co-surfactants were weighed out and then vortexed. They were placed at room temperature for 24 h, and the occurrence of stratification was observed. The stratification ratio was then removed. Unstratified prescription of 0.5 mL was weighed out and added to 100 mL of water, and this was left at 37 ± 2 °C for magnetic stirring to observe the formation of emulsification. With the surfactants, cosurfactants, and oil phases as one side, the proportion that could form clear and transparent oil droplets without floating was determined as the effective self-emulsification region in the phase diagram, and the ternary phase diagram was drawn accordingly. The composition of the SEDDS was as follows: CS, EL, and TP. The ternary phase diagram is shown in ***Figure 2***, with the black dots representing the test points and the area inside the black line representing the effective self-emulsification area. The results show that emulsification could be achieved within the scope of the investigation.

#### 3.2.2. The optimization of the prescription

##### 3.2.2.1. Optimizing the prescription of the Mixture-Optimal (Custom) design

The Mixture-Optimal (Custom) in Design Expert 11 software was used to optimize the composition in line with the ternary phase diagram. According to the scope of the investigation, the range of CS (A) was set within 20%–80%, that of EL(B) was set within 20%–80%, and that of TP (C) was set within 0%–30%. The artemisinin drug load, self-emulsification time, and emulsification time were taken as indicators for the design. The levels of experimental factors, design, and results are shown in ***Table 2***. The oleic acid polyethylene glycol glyceride, polyoxyethylene hydrogenated castor oil, and diethylene glycol monoethyl ether were weighed according to the central composite design table. Extra crude artemisinin was added for swirling and mixing, and the drug loading was determined. From each test site, 200 μL of the sample was taken and put into 2 mL of water. More crude artemisinin was added, and the mixture was shaken in a constant temperature oscillation box at 37 °C for 24 h. Then it was filtered, and the solubility was measured. At the same time, 0.2 mL of emulsion containing the above drugs was added to 200 mL of water at 37 °C in a dissolution tester. Self-emulsification was achieved by stirring the mixture slightly at a rotating speed of 50 rpm using the paddle method, and the emulsification time was recorded visually with a stopwatch.

**Table 2.** Design and results of Mixture-Optimal (Custom).

No.	A:CS	B:EL	C:TP	Drug loading (mg g^–1^)	Solutions (mg mL^–1^)	Time (s)
1	0.64	0.36	0	25.5335	3.0688	608.5
2	0.63	0.2	0.17	30.3141	2.7935	266
3	0.2	0.8	0	30.2070	0.8566	45.5
4	0.5	0.2	0.3	38.9640	1.7954	250
5	0.38	0.32	0.3	41.9705	1.5819	199.5
6	0.34	0.47	0.19	34.1621	1.6422	387.5
7	0.2	0.61	0.19	38.2159	1.2036	21
8	0.2	0.61	0.19	34.5575	1.1987	25
9	0.63	0.2	0.17	39.4521	2.0536	246.5
10	0.38	0.32	0.3	37.4445	1.5527	194.5
11	0.51	0.34	0.15	26.3412	2.2747	274.5
12	0.39	0.61	0	29.8002	1.4367	630
13	0.47	0.48	0.05	27.4283	1.9013	298
14	0.8	0.2	0	27.2061	2.9895	35
15	0.47	0.48	0.05	28.8753	2.1280	621
16	0.51	0.34	0.15	31.3770	1.7855	313

##### 3.2.2.2. Model fitting

Design Expert 11 software was used for the model fitting of the data. The regression equations of the fitting model were as follows: drug loading = 24.4728*A* + 29.9979*B* + 67.0748 *C*; solution = 3.7730*A* + 0.4282*B* + 0.0489*C*; time = –798.4724*A* − 801.8871*B* + 79.0604*C*  +  5613.8245 *A*×*B* + 1626.5823*A*×*C* − 956.45780*B*×*C*.

The fittings of each indicator are shown in ***Table 3***.

**Table 3.** Fitting table of various indicators.

Response	Model	Source	Sum of squares	df	Mean square	*F* value	*p* value	
Drug loading	Linear model	Model	290.35	2	145.18	15.06	.0004	Significant
		^(1)^Linear mixture	290.35	2	145.18	15.06	.0004	
		Residual	125.29	13	9.64			
		Lack of fit	52.88	8	6.61	0.4564	.8450	Not significant
		Pure error	72.41	5	14.48			
		Cor total	415.64	15				
Solutions	Linear model	Model	5.20	2	2.60	35.17	<.0001	Significant
		^(1)^Linear Mixture	5.20	2	2.60	35.17	<.0001	
		Residual	0.9606	13	0.0739			
		Lack of Fit	0.5411	8	0.0676	0.8061	.6263	Not significant
		Pure Error	0.4195	5	0.0839			
		Cor Total	6.16	15				
time	Quadratic model	Model	4.931 *E* + 05	5	98626.17	7.62	.0034	Significant
		^(1)^Linear Mixture	93897.03	2	46948.52	3.63	.0654	
		AB	3.714 *E* + 05	1	3.714E + 05	28.69	.0003	
		AC	3293.05	1	3293.05	0.2544	.6249	
		BC	1448.40	1	1448.40	0.1119	.7449	
		Residual	1.294 *E* + 05	10	12943.19			
		Lack of Fit	76315.64	5	15263.13	1.44	.3503	Not significant
		Pure Error	53116.25	5	10623.25			
		Cor Total	6.226 *E* + 05	15				

##### 3.2.2.3. Results

**Figure 3.**
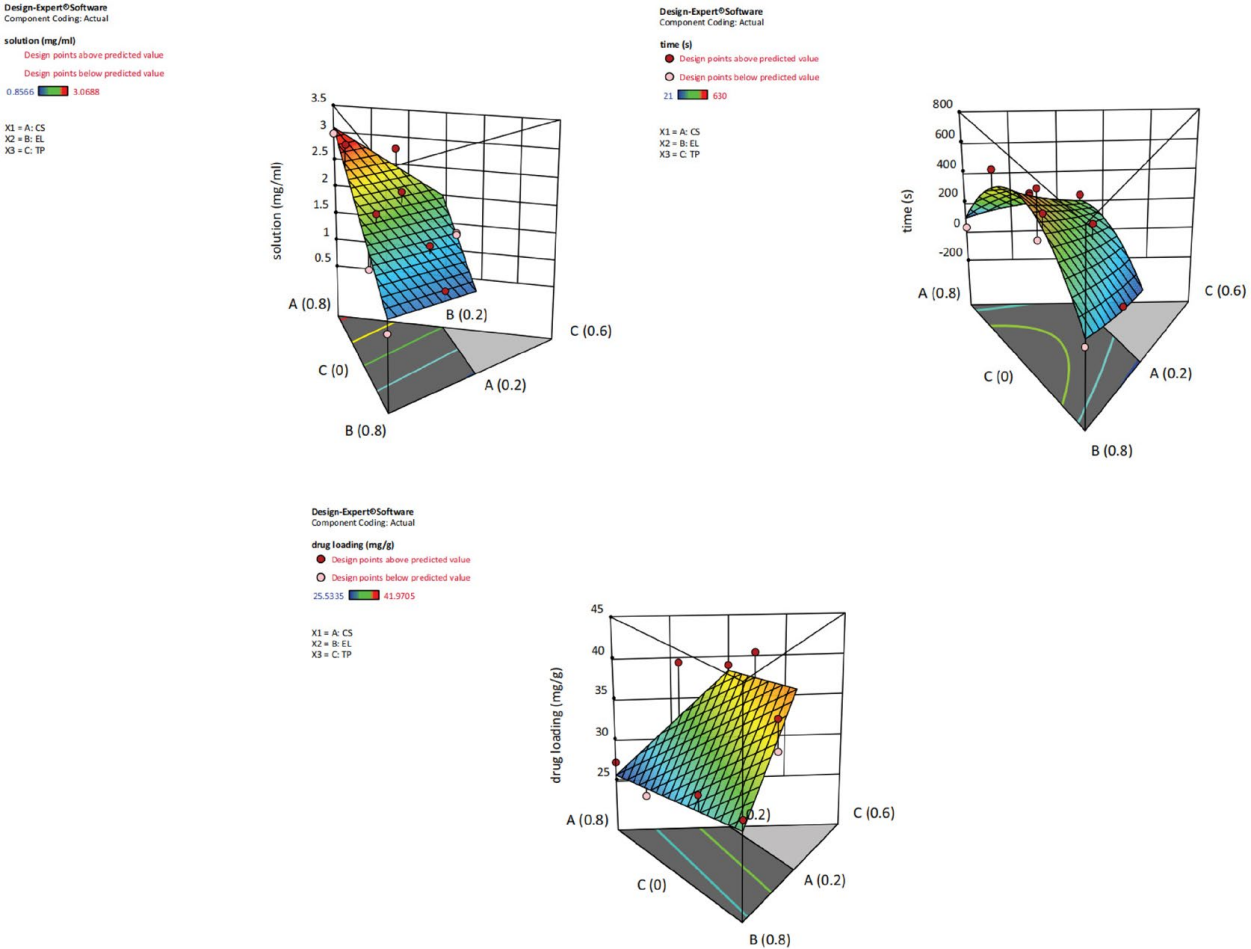
Contour map and response surface three-dimensional map of the prescription and indicators. Solution (mg/mL) is the effect surface diagram of the relationship between solubility and the dosage of A, B, and C. Time (s) is the effect surface diagram of the relationship between emulsification time and dosage of A, B, and C. Drug loading (mg/g) is the effect surface diagram of the relationship between drug loading amount and dosage of A, B, and C. According to the design requirements, the larger the solubility and drug load, and the smaller the emulsification time, the better the design is optimized.

The contour map and 3D-effect surface map concerning the three evaluation indicators and the influences of the three kinds of excipients obtained from the Mixture-Optimal (Custom) design are shown in ***Figure 3***. The composition of SEDDS prescription will be selected based on the solubility of artemisinin, and excipients with high solubility should be selected. Conditions such as non-stratification of three-phase excipients, the ability to form milk in contact with water, and large lactation area should also be met. This study has investigated more than a dozen combinations of prescriptions, some of which have stratification, some of which are not emulsifying, etc., and finally, only this combination has no stratification and large lactation area within the scope of investigation.

##### 3.2.2.4. The prediction and validation of the optimal prescription

In the present study, the prescription composition of artemisinin self-emulsification preparation was optimized with a large drug load, maximum solubility, and the shortest self-emulsification time. The predicted optimal prescription ratio was as follows: CS:EL:TP = 0.5:0.2:0.3 (wt/wt/wt). The predicted drug load was 38.358 mg/g, the solubility was 1.987 mg/mL, and the self-emulsification time was 212 s.

The self-emulsifying prescription was prepared with the predicted optimal formula ratio, and the results showed that the detected drug load was 41.556 mg/g, the solubility was 1.997 mg/mL, and the self-emulsification time was 231 s. The absolute deviation of each indicator was less than 10%, which confirmed the sound prediction of the mathematical model.

### 3.3. The determination of the apparent oil–water partition coefficient

#### 3.3.1. The determination method

The shaking flask method was used for the determination (Lin et al., 2013; Bao et al., 2016). An extra amount of artemisinin was dissolved in water, hydrochloric acid solution with pH 1.2, and phosphate-buffer saturated *n*-octanol with a pH of 4.5, 6.8, or 7.4 and put into a saturated solution of *n*-octanol. Then 1.0 mL of the solution was measured out precisely and put into five plug test tubes. A 4 mL volume of water saturated with *n*-octanol and the corresponding pH buffer were added successively. After being vortexed for 5 min and shaken in a constant-temperature oscillation incubator at (37 ± 2)°C for 24 h, the tubes were taken out and left to stand for 30 min. Then the tubes were centrifuged at 10,000 rpm for 10 min to separate the two phases. The water layer and alcohol layer were then taken for detection. The concentration of artemisinin and the logarithm of apparent permeability (log *P*_app_) were calculated using the following formula:

$$p_{\text{app}}=\frac{C_{\text{oil}}}{C_{\text{water}}}=\frac{4\times (C_{t})}{C_{0}-C_{t}}$$

In the above equation, *p*_app_ is the apparent oil/water distribution coefficient, *C*_0_ is the initial concentration of the drug in the *n*-octanol, and *C_t_* is the concentration measured in the oil phase when the distribution of the drug reaches equilibrium.

#### 3.3.2. The results of the oil–water distribution coefficient

As shown in ***Table 4***, the log *p*_app_ value of the crude artemisinin was between 0 and 3 when water was used as the medium. The log *p*_app_ values of the crude drug were all less than 0 when other pH buffers were used as the medium. In the solutions where different pH values were used as the medium, the log *p*_app_ of the artemisinin self-emulsifying preparation was within the range of 0–3, indicating that the artemisinin self-emulsifying preparations were more absorbable by the body than the crude drug.

**Table 4.** Results of apparent oil-water distribution coefficient of artemisinin crude drug.

Solvent	Crude drug		SEDDS	
	*p*_app_	log*p*_app_	*p*_app_	log*p*_app_
Distilled water	149.1152	2.1735	339.7131	2.5311
Hydrochloric acid solution pH 1.2	–12.2299	–1.0874	226.4715	2.3550
phosphate buffer pH 4.5	–69.3641	–1.8411	150.7829	2.1784
phosphate buffer pH 6.8	–28.3678	–1.4528	161.1476	2.2072
phosphate buffer pH 7.4	–124.1285	–2.0939	229.2047	2.3602

#### 3.3.3. The results of scanning electron microscopy before and after artemisinin addition

**Figure 4.**
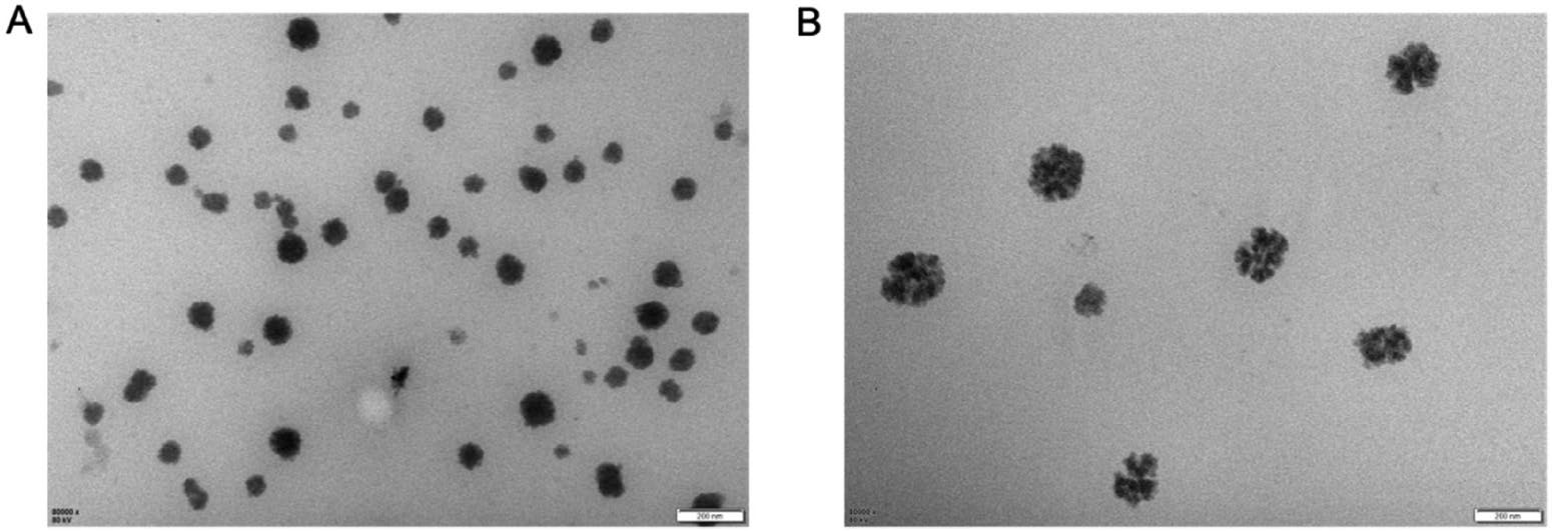
Scanning electron microscopy before (A) and after artemisinin addition (B). There was no significant change in emulsifying time before and after artemisinin addition. The appearance was clear and transparent before and after artemisinin was added. Particle size: The particle size and potential of SEDDS without artemisinin were not measured. The average size of the particle with artemisinin was 128.0 nm, PdI: 0.422, and the average zeta was –4.29.

As shown in ***Figure 4***, there was no significant change in emulsifying time before and after artemisinin addition. The appearance was clear and transparent before and after artemisinin was added. The particle size and the potential of SEDDS without artemisinin were not measured. The average size of the particle with artemisinin was 128.0 nm, the polydispersity index (PdI) was 0.422, and the average electrokinetic potential (zeta) was –4.29 mV.

### 3.4. The comparison of bioavailability

#### 3.4.1. The administration protocol and blood sample collection

Ethics approval was obtained for the animal experiments in this study (ethics approval no. 20060801). Twelve specific pathogen-free grade male jugular vascular catheterization (JVC) rats weighing 200 ± 20 g and fasted for 12 h before the experiment (with free drinking) were randomly divided into two groups (six in each group). Artemisinin SEDDS was administered to one group of rats at 40 mg/kg, and crude artemisinin was administered to the other group at an equivalent amount of 40 mg/kg. Blood samples were collected at 10, 30, 60, 90, 120, 240, 360, and 480 min after administration. Each time 50 μL of plasma was separated out and added to 400 μL of methanol-acetonitrile (1:1, vol/vol) solution with caffeic acid as the internal standard. This was followed by vortex mixing for 2 min and centrifugation at 12,000 rpm for 15 min, after which 400 μL of the supernatant was put into an Eppendorf tube and blown dry with nitrogen. Then 50 μL of the methanol–acetonitrile (1:1, vol/vol) was ultrasonically re-dissolved for 3 min and centrifuged at 12,000 rpm for 15 min. A 3 μL volume of supernatant was then taken for analysis.

#### 3.4.2. The relative bioavailability

Artemisinin SEDDS and crude artemisinin were administered to the two groups of rats in a single intragastric dose. DAS 3.0 pharmacokinetic software analysis (the detailed pharmacokinetic data will be published separately) was used to compare the bioavailability (***Table 5***).

**Table 5.** The pharmacokinetic parameters of the intragastric administration of gavage fluid containing the artemisinin SEDDS and crude drug, respectively in rats (*n* = 6).

Parameters	Artemisinin crude drug	Artemisinin SEDDS
AUC 0→12 (μg/L min)	3,016,425	4,459,035.05
AUC 0→∞ (μg/L min)	3,041,632.674	4,521,064.234
AUMC 0→12 (min min μg/L)	1,027,814,760	1,021,461,252
AUMC 0→∞ (min min μg/L)	1,043,659,762	1,072,529,531
MRT 0→48 (min)	340.739	252.59375
MRT 0→∞ (min)	343.125	259.57175
VRT 0→48 (min^2^)	4,144.697	14,908.2695
VRT 0→∞ (min^2^)	4,974.244	20,090.61025
*T*_max_ (min)	360	180
*C*_max_ (μg/L)	22,640	48,691.25

It was found that the SEDDS could improve the relative oral bioavailability of artemisinin. The relative bioavailability of artemisinin SEDDS at the same dosage was 1.47 times greater than that of the intragastric administration of the crude drug. At the same time, the SEDDS accelerated the peak time of the blood concentration of the drug.

## 4. Discussion

In creating the artemisinin self-emulsifying preparations, the combination of the oil phase, surfactants, and co-surfactants with the maximum solubility of the crude drug was preferred (Nishida et al., 2010). However, as the ternary phase diagram in the three phases failed to achieve the formation of emulsification, the non-maximum solubility of the ingredients was selected.

The optimal prescription obtained by the Design Expert 11 software was the same as the fourth design point in the design process, but there was a large difference between the results. The reason might be that the sum of all the weighed samples was 1 g in the design process, and the total amount of weighed samples increased when making the optimal prescription, leading to different prescription results with the same weighing sample error (Sun, 2006).

In determining the oil and water distribution coefficient, the effects of different pH buffers on the crude artemisinin and the self-emulsifying preparations were investigated. Theoretically, the log *p*_app_ value reflects the lipophilicity and hydrophilia of the drug, and the larger the log*p*_app_ value, the higher the lipophilicity, and the lower the log*p*_app_ value, the higher the hydrophilia. When log *p*_app_ < 0, it is very difficult for the gastrointestinal tract to absorb the drug, whereas when 0 < log*p*_app_ < 3 the drugs can be absorbed by the gastrointestinal tract. When log*p*_app_ > 3, it means that the drugs have strong lipid solubility, and this is not conducive to gastrointestinal absorption (Nishida et al., 2010; Sun, 2006). Drugs need to have both a certain degree of water solubility and fat solubility so as to be better absorbed by the body. This is because, in addition to the aqueous environment, a variety of oils and fats in the body are also involved in the absorption of drugs. Hence, the high water-solubility or fat-solubility of drugs is not conducive to their absorption (Nishida et al., 2010; Sun, 2006). The results showed that the body did not easily absorb crude artemisinin, although the log*p*_app_ values of the crude artemisinin were moderate when water was adopted as the medium. Since different body fluids with different pH values exist in the body, the water distribution coefficient predicted by different pH buffers might be more meaningful (Fernández-Pumarega et al., 2020). In particular, the pH values of different parts of the human digestive system are varied and could provide guidance for the study of artemisinin absorption and its distribution *in vivo.* It could also provide a basis for the study of artemisinin formulations (Hoyle, 1997). At present, the mechanism of SEDDS formation is not clear, and there is no clear academic report. Relatively mature interfacial tension theory, micellar theory, thermodynamic theory, and so on. Zeta potential was the result of determination of SEDDS prepared by adding water, and the stability of SEDDs without adding water was better. Stability studies have been carried out at 60 °C closed for 30 days, no stratification was observed, and no significant change in drug loading. .

This study is currently in the *in vitro* phase and has not entered the stage of safety evaluation. Excessive use of SEDDS has been reported to be associated with adverse effects, so this study optimized the recertification system for maximum solubility and reduced the use of SEDDS excipients. The specific clinical usage of excipients will be predicted according to the bioavailability in the body. At present, artemisinin and artemisinin derivatives used in clinical practice are directly prepared as solid preparations or combined drugs (Zhang et al., 2015). Compared to these agents, the artemisinin SEDDS in this study increased the relative oral bioavailability and reduced the dosage. However, in this study, the toxicity of the SEDDS was not evaluated, and this part of the study will be carried out in the future.

The results of the *in vivo* pharmacokinetic study showed that besides artemisinin, other metabolites like dihydroartemisinin and artesunate were also present. In the rats treated with artemisinin API, the peak time of plasma concentration was longer and the duration of action was shorter. In addition, the peak time of artemisinin SEDDS was shorter and the duration was longer, and there was a double-peak phenomenon. The detailed metabolism of artemisinin *in vivo* needs further study. As artemisinin is a first-line antimalarial drug, improving its bioavailability can reduce the dosage required. This is of great significance for resource conservation and environmental protection.

## Data Availability

All data generated or analyzed during this study are included in this article. Further enquiries can be directed to the corresponding author.
